# 

**Published:** 1892-04

**Authors:** 


### CONTENTS FOR APRIL, 1892.

ORIGINAL COMMUNICATIONS.

Report of a Case of Double Dislocation of the Knee-Joints. By THOMAS H. MANLEY, M. D. ..... 513

CLINICAL REPORTS.

Sub-Acute Parenchymatous Hepatitis and Gastro-Enteritis. By A. L. BENEDICT, A. M., M. D. ..... 522

SOCIETY PROCEEDINGS.

New York Academy of Medicine—Section on Orthopedic Surgery ..... 524
Medical Society of the County of Erie ..... 532

PROGRESS IN MEDICAL SCIENCE.

Therapeutic Notes ..... 539

CORRESPONDENCE.

Berlin Letter ..... 543

EDITORIAL.

The Buffalo Academy of Medicine ..... 549
The New York Physician's Mutual Aid Association ..... 550
The Pan-American Medical Congress ..... 551
Editor's Drawer ..... 551

PERSONAL.

Dr. E. E. Montgomery—Dr. Joseph Price—Dr. Richard Curran ..... 554
Dr. John C. Fisher—Dr. James F. W. Ross—Dr. John A. Ouchterlony ..... 555

OBITUARY.

Dr. John Davidson Hill ..... 556

SOCIETY MEETINGS.

The American Medical Association ..... 556
The First Pan-American Medical Congress ..... 556
An International Periodical Congress of Gynecology and Obstetrics ..... 557

Book Reviews ..... 557
Miscellany ..... 569

